# Cu, Fe, and Zn isotope ratios in murine Alzheimer's disease models suggest specific signatures of amyloidogenesis and tauopathy

**DOI:** 10.1016/j.jbc.2021.100292

**Published:** 2021-01-14

**Authors:** Nikolay Solovyev, Ahmed H. El-Khatib, Marta Costas-Rodríguez, Karima Schwab, Elizabeth Griffin, Andrea Raab, Bettina Platt, Franz Theuring, Jochen Vogl, Frank Vanhaecke

**Affiliations:** 1 Department of Chemistry, Atomic & Mass Spectrometry–A&MS Research Unit, Ghent University, Ghent, Belgium; 2 BAM Bundesanstalt für Materialforschung und –prüfung, Division 1.1 Inorganic Trace Analysis, Berlin, Germany; 3 Department of Pharmaceutical Analytical Chemistry, Faculty of Pharmacy, African Union Authority St, Abbassia, Ain Shams University, Cairo, Egypt; 4 Institute of Medical Sciences, School of Medicine, Medical Sciences & Nutrition, Foresterhill, University of Aberdeen, Aberdeen, Scotland, United Kingdom; 5 Trace Element Speciation Laboratory (TESLA), Department of Chemistry, University of Aberdeen, Aberdeen, Scotland, United Kingdom; 6 Institute of Chemistry, Environmental Analytical Chemistry, University of Graz, Graz, Austria; 7 Charité – Universitätsmedizin Berlin, Institute of Pharmacology, Berlin, Germany

**Keywords:** Alzheimer’s disease, tau, amyloid-beta, copper, iron, zinc, multicollector inductively coupled plasma–mass spectrometry (ICP-MS), brain, serum, isotopic analysis, AD, Alzheimer’s disease, APP, amyloid precursor protein, BBB, blood–brain barrier, CSF, cerebrospinal fluid, FTD, frontotemporal dementia, ICP-MS, inductively coupled plasma–mass spectrometry, MC-ICP-MS, multicollector sector field inductively coupled plasma–mass spectrometry, WT, wild-type

## Abstract

Alzheimer’s disease (AD) is characterized by accumulation of tau and amyloid-beta in the brain, and recent evidence suggests a correlation between associated protein aggregates and trace elements, such as copper, iron, and zinc. In AD, a distorted brain redox homeostasis and complexation by amyloid-beta and hyperphosphorylated tau may alter the isotopic composition of essential mineral elements. Therefore, high-precision isotopic analysis may reveal changes in the homeostasis of these elements. We used inductively coupled plasma–mass spectrometry (ICP-MS)-based techniques to determine the total Cu, Fe, and Zn contents in the brain, as well as their isotopic compositions in both mouse brain and serum. Results for male transgenic tau (Line 66, L66) and amyloid/presenilin (5xFAD) mice were compared with those for the corresponding age- and sex-matched wild-type control mice (WT). Our data show that L66 brains showed significantly higher Fe levels than did those from the corresponding WT. Significantly less Cu, but more Zn was found in 5xFAD brains. We observed significantly lighter isotopic compositions of Fe (enrichment in the lighter isotopes) in the brain and serum of L66 mice compared with WT. For 5xFAD mice, Zn exhibited a trend toward a lighter isotopic composition in the brain and a heavier isotopic composition in serum compared with WT. Neither mouse model yielded differences in the isotopic composition of Cu. Our findings indicate significant pathology-specific alterations of Fe and Zn brain homeostasis in mouse models of AD. The associated changes in isotopic composition may serve as a marker for proteinopathies underlying AD and other types of dementia.

Alzheimer’s disease (AD) is the most common cause of dementia, accounting for about two-thirds of the currently reported 50 million dementia cases worldwide. By 2050, about 152 million people are likely to be diagnosed with dementia. With a current cost of about a trillion US dollars a year (expected to double by 2030) and being a major cause of death, dementia is a growing global health concern that places a significant burden on societies and healthcare systems. Therefore, there is an urgent need to develop interventions and treatments to reverse or at least slow down the progression of AD (1, 2, 3). As early and specific diagnosis is essential for effective therapeutics, current research efforts also focus on the discovery of biomarkers (2, 3) enabling disease detection during early stages (4).

The pathology of AD involves the misprocessing of the amyloid precursor protein (APP), which results in the accumulation and buildup of soluble and fibrillar amyloid-beta (Aβ) and other metabolites (5). Additionally, hyperphosphorylated tau, a microtubule-associated protein, leads to the formation of neurofibrillary tangles, composed of a truncated 100-amino acid fragment of tau (6), which can autonomously catalyze the conversion of normal soluble tau into tau fibrils and tau aggregates (7). Both tau and Aβ aggregation contribute to AD pathology, but hypotheses differ as to which of these is the primary causative factor (8). Nevertheless, both are hallmarks of AD used for the ultimate postmortem confirmation of AD (4).

Previous observations of hot spots of certain metals alongside tau/Aβ accumulation suggested that spatial or even mechanistic correlations exist between tau/Aβ and certain trace elements. Essential trace elements such as copper (Cu), iron (Fe), and zinc (Zn) have fundamental physiological roles in, *e.g.*, enzymatic reactions, oxygen transport, and cellular signaling; their homeostasis is therefore crucial for proper functioning of the brain (9, 10, 11). Furthermore, elevated Cu, Fe, and Zn levels were found in Aβ plaques in AD brain tissues (12, 13, 14, 15), suggesting a direct or indirect involvement of trace metals in AD pathogenesis. Recent systematic reviews indicated weak associations between Cu, Fe, and Zn and AD, with some studies reporting increased levels of these metals, while others are reporting decreased levels in the media investigated (predominantly blood and to a lesser extent cerebrospinal fluid (CSF), nails, and hair) of AD patients (16, 17). However, more mechanistic studies indicate several possible pathways relating Cu, Fe, and Zn with AD pathology (18, 19, 20).

To date, both tau- and Aβ-based animal models are widely used in AD research. Several tau transgenic mouse models have been generated, most of them based on overexpression of mutant tau (21, 22), though it is important to note that these mutations are based on frontotemporal dementia (FTD) and not AD. Lines include, for example, the P301L mouse, which overexpresses the aforementioned mutation in the longest human tau isoform (htau40). Expression of P301L htau40 results in early deposition of tau tangles, gliosis, axonal degeneration, and motor and behavioral deficits (23).

Line 66 (L66) mice express full-length human tau with the P301S mutation under the control of the Thy1-regulatory element (24). The P301S mutation has been previously associated with tau aggregation (25, 26, 27). L66 mice overexpress the longest human tau isoform (htau40) with 441 amino acid residues, under the control of the mouse *Thy1*-promoter. These mice show early onset high tau load in hippocampal and cortical neurons (24) and robust inflammation in both forebrain and hippocampal system (28) reminiscent of the behavioral variant of FTD with tau pathology. The L66 murine model has widely abundant tau pathology throughout the brain, with particularly high tau aggregation in neurons of the hippocampus and entorhinal cortex, eventually leading to neuronal loss (24). Behaviorally, these mice are characterized by abnormal gait pattern and dysfunction in motor coordination and motor learning as early as 4 to 5 weeks of age (24).

As for Aβ models, first attempts to generate AD-like pathology in mice by overexpressing APP were only partly successful, as mice tended to produce only low Aβ-associated pathology and often failed to show behavioral impairments (29, 30). Later, the familial AD model (5xFAD) was created by combining five mutations related to human APP and presenilin (an enzyme converting APP to Aβ (23)), which are linked to autosomal dominant forms of familial AD (FAD) (31). The 5xFAD mice are double transgenic for APP and PSEN1 with a total of five AD-linked mutations: the Swedish (K670N/M671L), Florida (I716V), and London (V717I) mutations in the APP gene, as well as the M146L and L286V mutations in the PSEN1 gene. These mutations lead to accelerated Aβ plaque formation and deposition and eventually to neuronal loss and working memory impairments (32, 33). These mice are characterized by aggressive Aβ neuropathology and early behavioral deficits.

Both Line 66 and the 5XFAD models have been extensively characterized in terms of pathology and cognition (24, 32, 33) and were used in the current study for brain and serum analysis. We have used quadrupole-based and sector field inductively coupled plasma–mass spectrometry (ICP-MS) for quantification of the total element contents of Cu, Fe, and Zn and multicollector sector field inductively coupled plasma–mass spectrometry (MC-ICP-MS) for measuring their isotope ratios (expressed as delta (*δ*) values) and demonstrated the traceable and precise determination of the total element contents and isotope ratios of Cu, Fe, and Zn.

Mutant tau expressing mice showed a lighter isotopic composition of Fe (enriched in the lighter isotopes) in the brain and to a lower extent in blood serum, as well as higher Fe contents in the brain than matched wild-type (WT) mice. For the 5xFAD mice compared with controls, a trend toward a lighter Zn isotopic composition was observed in the brain tissue and blood serum. The results of this study may provide a step forward concerning the potential use of the Cu, Fe, and Zn isotopic information for diagnostic purposes and/or to achieve a more profound understanding of AD.

## Results

Analytical methods applied in the different research facilities involved were evaluated and thereafter implemented for the traceable quantitative determination and for the accurate and precise isotopic analysis of Cu, Fe, and Zn in the brain tissue and blood serum of L66 and 5xFAD mice, as well as of their respective controls. The measurements undertaken and research facilities responsible are summarized in Table 1. Only SI-traceable data (here mass fractions) or data being traceable to the same internationally accepted source (accomplished *via* the use of delta values against internationally accepted isotopic reference materials) are metrologically comparable. The total elemental content quantification was validated between BAM and the University of Aberdeen; isotope ratio measurements were validated between BAM and Ghent University.

**Table 1:** Mouse groups and cohort sizes (n) used to measure total Fe, Cu, and Zn contents in the brain tissue as well as their isotopic composition in brain and serum samples

Housing facility	Mouse line	Tissue	Metal	n	Analytical research facility
Charité - Berlin	L66^a^	Brain	Total Fe	13	University of Aberdeen (n = 13)
			Total Cu	26	BAM (n = 13) and University of Aberdeen (n = 13)
			Total Zn	26	BAM (n = 13) and University of Aberdeen (n = 13)
			IR Fe	13	Ghent University (n = 13)
			IR Cu	26	BAM (n = 13), Ghent University (n = 13)
			IR Zn	26	BAM (n = 13), Ghent University (n = 13)
		Serum	IR Fe	13	Ghent University (n = 13)
			IR Cu	24	BAM (n = 11), Ghent University (n = 13)
			IR Zn	24	BAM (n = 11), Ghent University (n = 13)
Charité - Berlin	NMRI-WT^a^	Brain	Total Fe	5	University of Aberdeen (n = 5)
			Total Cu	11	BAM (n = 6), University of Aberdeen (n = 5)
			Total Zn	11	BAM (n = 6), University of Aberdeen (n = 5)
			IR Fe	5	Ghent University (n = 5)
			IR Cu	11	BAM (n = 6), Ghent University (n = 5)
			IR Zn	11	BAM (n = 6), Ghent University (n = 5)
		Serum	IR Fe	5	Ghent University (n = 5)
			IR Cu	9	BAM (n = 4), Ghent University (n = 5)
			IR Zn	9	BAM (n = 4), Ghent University (n = 5)
University of Aberdeen	5xFAD^a^	Brain	Total Fe	8	University of Aberdeen (n = 8)
			Total Cu	18	BAM (n = 10), University of Aberdeen (n = 8)
			Total Zn	18	BAM (n = 10), University of Aberdeen (n = 8)
			IR Fe	10	Ghent University (n = 10)
			IR Cu	20	BAM (n = 10), Ghent University (n = 10)
			IR Zn	20	BAM (n = 10), Ghent University (n = 10)
		Serum	IR Fe	9^b^	Ghent University (n = 9)
			IR Cu	14^b^	BAM (n = 5), Ghent University (n = 9)
			IR Zn	9^b^	Ghent University (n = 9)
University of Aberdeen	BL6-WT^a^	Brain	Total Fe	9	University of Aberdeen (n = 9)
			Total Cu	19	BAM (n = 10), University of Aberdeen (n = 9)
			Total Zn	19	BAM (n = 10), University of Aberdeen (n = 9)
			IR Fe	10	Ghent University (n = 10)
			IR Cu	20	BAM (n = 10), Ghent University (n = 10)
			IR Zn	20	BAM (n = 10), Ghent University (n = 10)
		Serum	IR Fe	8^b^	Ghent University (n = 8)
			IR Cu	14^b^	BAM (n = 6), Ghent University (n = 8)
			IR Zn	8^b^	Ghent University (n = 8)

IR, isotope ratio.

Details for mouse housing facilities, as well as for facilities conducting MS measurements are indicated. All animals were males.

a IR analysis of mouse chow was conducted at Ghent University.

b Reduced mouse number included in the analyses due to failed sampling.

Given that diet is the major source of metal exposure and that mouse groups received different chows in the different housing facilities, the animals’ chow was also analyzed for its Cu, Fe, and Zn isotopic compositions. L66 mice and their NMRI wild-type (NMRI-WT) controls (housed at Charité) received the same chow (V1534-3) for the first 10 months, but between 10 and 12 months (time of sacrifice) L66 received a different chow with higher protein content (V1124–3) because they developed a considerable tremor. Total contents of Cu, Fe, and Zn in both types of chow were in line with the manufacturers’ data. However, their *δ*^65^Cu, *δ*^66^Zn, *δ*^67^Zn, and *δ*^68^Zn values were significantly different from each other (Table S1). Conversely, 5xFAD mice and their C57BL6/J wild-type (BL6-WT) controls received the same chow during the whole experimentation period; the total contents of Cu, Fe, and Zn in this chow were in line with the manufacturers’ data.

### Total Cu, Fe, and Zn levels in mouse brain

The total contents of Cu, Fe, and Zn in mouse brain (per wet tissue weight) were determined by sector field and quadrupole-based inductively coupled plasma–mass spectrometry (SF-ICP-MS and Q-ICP-MS, respectively). Since the amount of serum collected was insufficient for accurate quantification, only isotopic analysis was conducted (see below).

**Figure 1:**
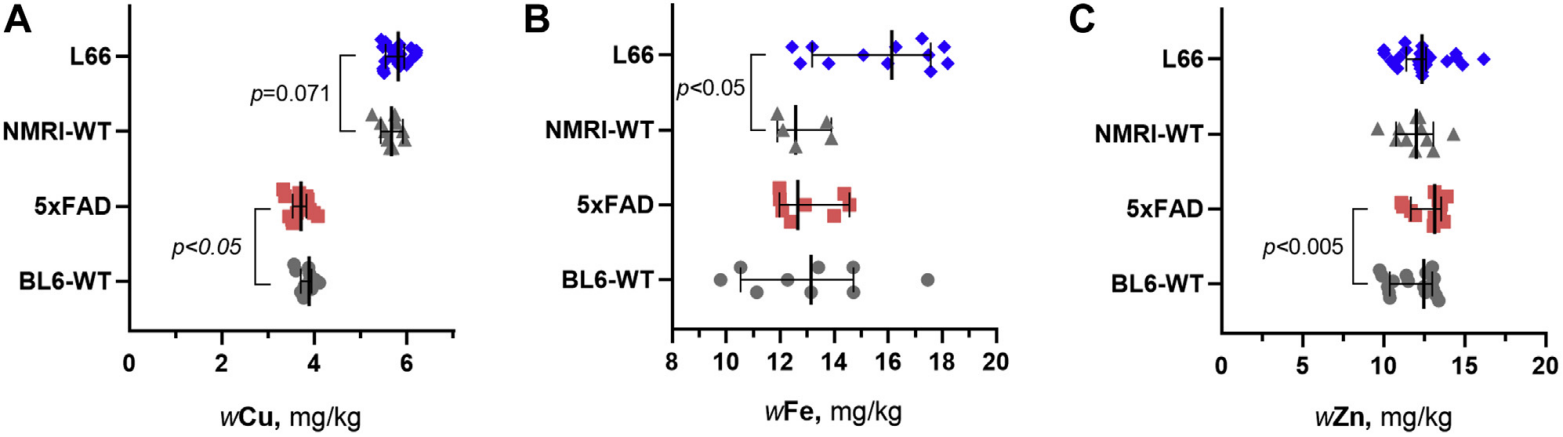
**Cu (*A*), Fe (*B*), and Zn (*C*) contents (in mg per kg of wet tissue) in the brain tissue of L66 and 5xFAD mice relative to matched WT-controls.** Values are presented as dot scatter plots showing the median with error bars indicating the 95% CI. Total element contents were measured by ICP-MS. Statistical analysis was conducted using ANCOVA (*A* and *C*) and *t*-tests (*B*). All animals were males; the age of the animals was 11 to 12 months and 5 to 6 months for L66/NMRI-WT and 5xFAD/BL6-WT, respectively. Detailed data are presented in Table S2. The numbers of animals analyzed at the different research facilities are indicated in Table 1.

Quantitative determination of the elements of interest in the brain tissue indicated that L66 mice had higher contents of Fe than NMRI-WT (*p* < 0.05, see Fig. 1 and Table S2*A*). Also for Cu and Zn, a higher content was observed in the brain tissue of L66 mice, though the increase was not statistically significant (*p* = 0.071 and *p* = 0.455, respectively). *Post-hoc* analysis (the chow was analyzed along with the mice brain and serum) of the metal contents in the diet of the animals did not suggest that differences in the metal contents of the diets were at the origin of these observations. Element quantification of 5xFAD brains (Fig. 1 and Table S2*B*) indicated significantly *lower* levels of Cu (*p* < 0.05), but a higher content of Zn in brain tissue of 5xFAD mice compared with BL6-WT (*p* < 0.01).

### Isotopic signatures of Cu, Fe, and Zn in mouse brain and blood serum

**Figure 2:**
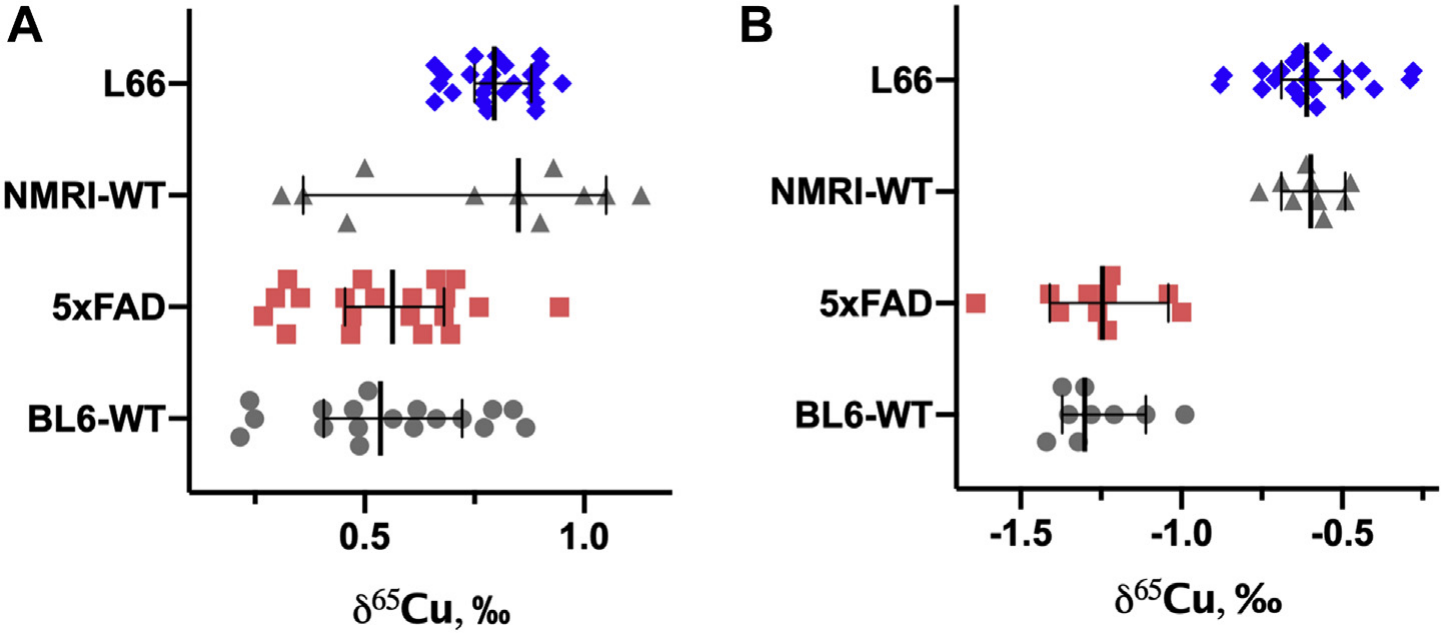
**Isotopic signatures of copper (*δ***^**65**^**Cu) in the brain (A) and blood serum (B) of L66 *versus* NMRI-WT and 5xFAD *versus* BL6-WT mice.** Values are presented as dot scatter plots showing the median with error bars indicating the 95% CI. Isotope ratios were measured by MC-ICP-MS. Statistical analysis was conducted using ANCOVA. Detailed data are presented in Tables S3 and S4. All animals were males; the age of the animals was 11 to 12 months and 5 to 6 months for L66/NMRI-WT and 5xFAD/BL6-WT, respectively. The numbers of animals analyzed at the different research facilities are indicated in Table 1.

**Figure 3:**
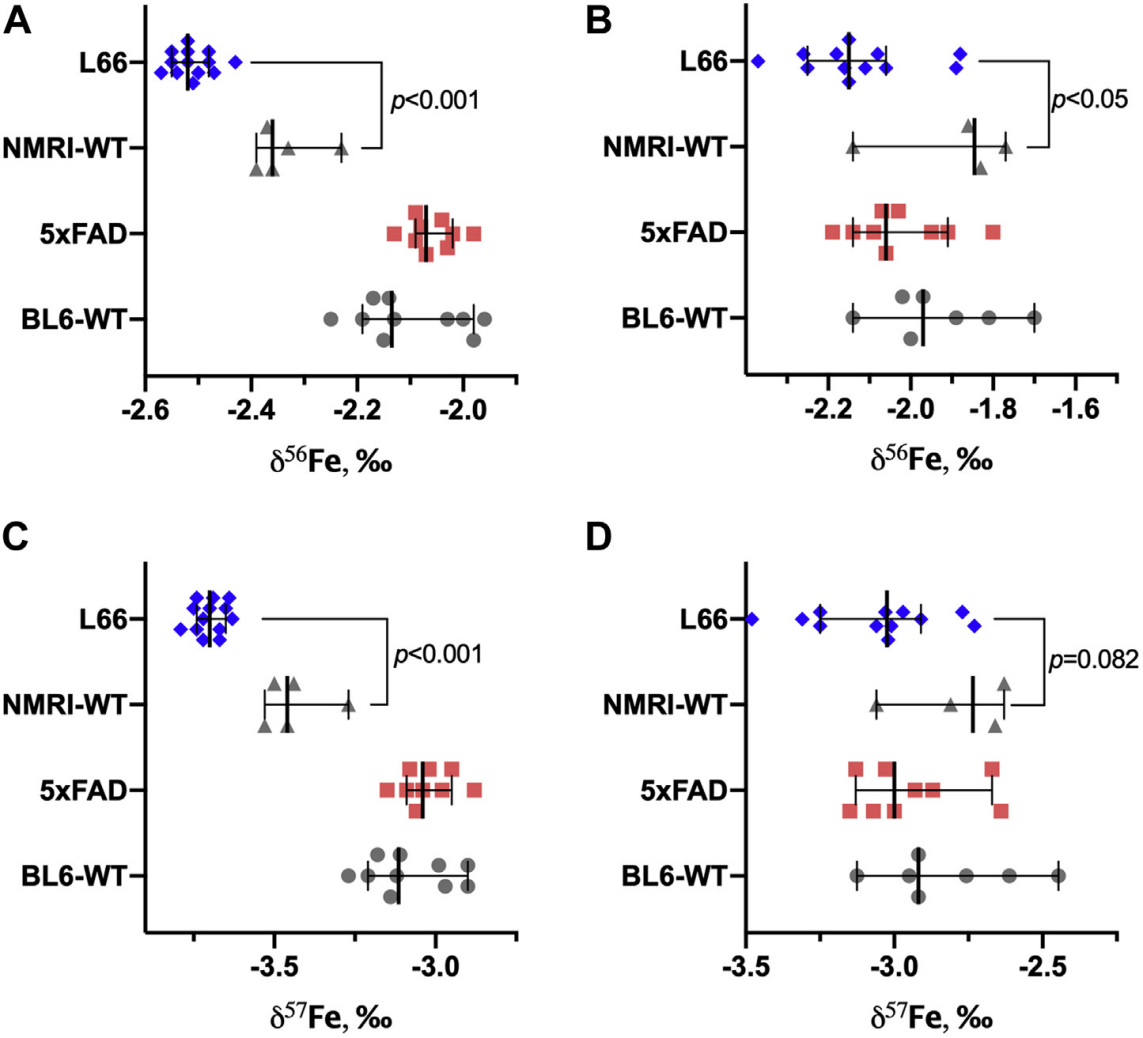
**Isotopic signatures of iron (*δ***^**56**^**Fe—*A* and *B*; *δ***^**57**^**Fe—*C* and *D*) in the brain (*A* and *C*) and blood serum (*B* and *D*) of L66 *versus* NMRI-WT and 5xFAD *versus* BL6-WT mice.** Values are presented as dot scatter plots showing the median with error bars indicating the 95% CI. Isotope ratios were measured by MC-ICP-MS. Statistical analysis was conducted using a Mann–Whitney rank test. Detailed data are presented in Tables S3 and S4. All animals were males; the age of the animals was 11 to 12 months and 5 to 6 months for L66/NMRI-WT and 5xFAD/BL6-WT, respectively. The numbers of animals analyzed at the different research facilities are indicated in Table 1.

**Figure 4:**
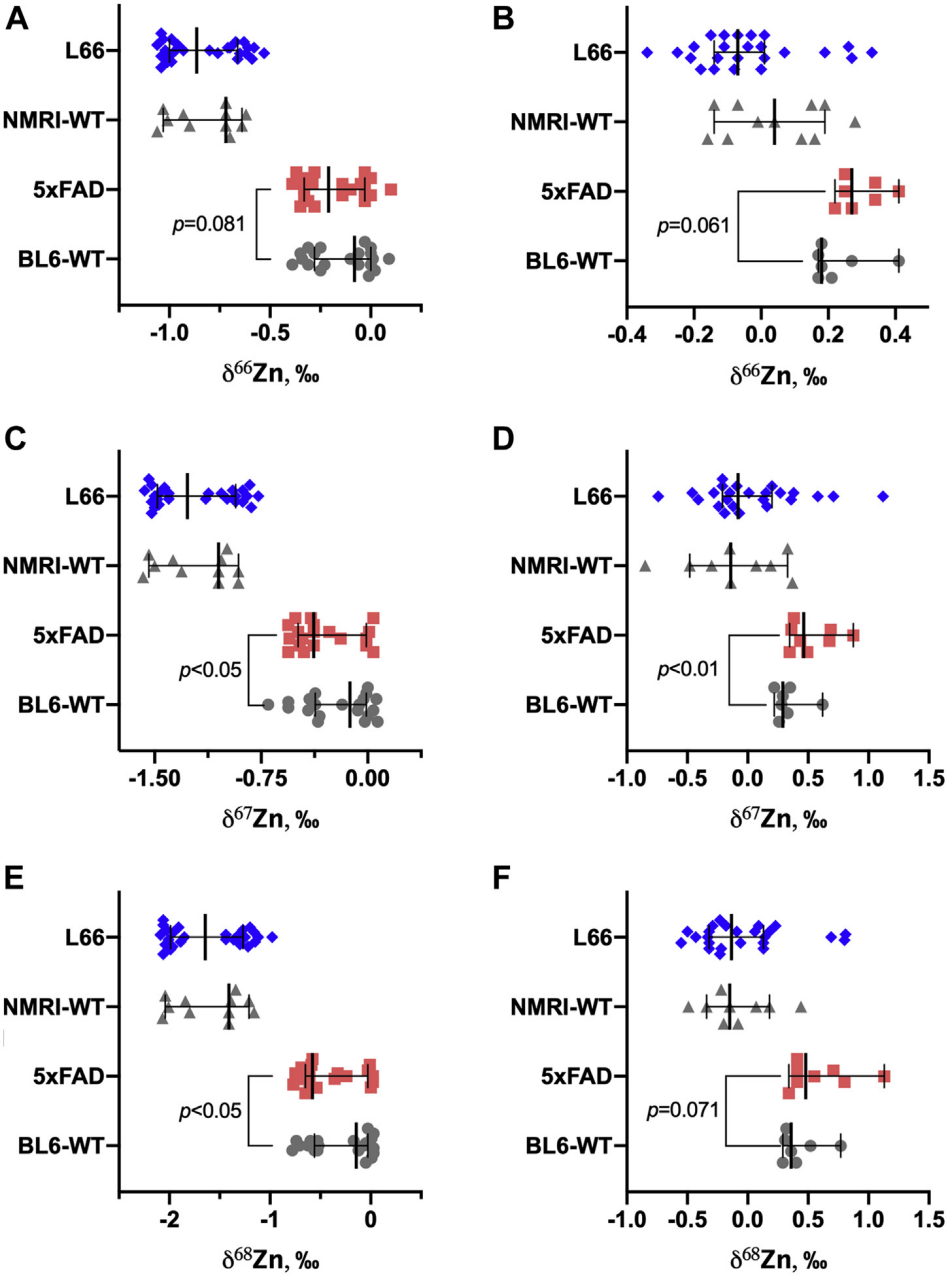
**Isotopic signatures of zinc (*δ***^**66**^**Zn—*A* and *B*; *δ***^**67**^**Zn—*C* and *D*; *δ***^**68**^**Zn—*E* and *F*) in the brain (*A*, *C*, and *E*) and blood serum (*B*, *D*, and *F*) of L66 *versus* NMRI-WT and 5xFAD *versus* BL6-WT mice.** Values are presented as dot scatter plots showing the median with error bars indicating the 95% CI. Isotope ratios were measured by MC-ICP-MS. Statistical analysis was conducted using ACOVA and a Mann–Whitney rank test (only for 5xFAD and BL6-WT in *B*, *D*, and *F*). Detailed data are presented in Tables S3 and S4. All animals were males; the age of the animals was 11 to 12 months and 5 to 6 months for L66/NMRI-WT and 5xFAD/BL6-WT, respectively. The numbers of the animals analyzed at the different research facilities are indicated in Table 1.

The isotopic compositions of Cu, Fe, and Zn in the brain tissue and blood serum were compared at group levels and the results obtained, expressed as *δ-*values, are presented in Figures 2, 3 and 4 (detailed data in Tables S3 and S4). Additionally, for all samples analyzed, the individual *δ*-values are provided in the Supplementary Data file.

For the brain of L66 compared with that of the NMRI-WT mice, the *δ*^56^Fe and *δ*^57^Fe values (*p* < 0.001) indicated a significantly lighter Fe isotopic composition (enrichment in the lighter ^54^Fe isotope), and this shift was partially confirmed for serum levels (*p*= 0.02 and 0.082 for *δ*^56^Fe and *δ*^57^Fe, respectively) – Figure 3 (Tables S3*A* and S4*B* for brain and serum, respectively). The absolute shift between the L66 and NRMI was found to be *Δ*^56^Fe = −0.16‰ and *Δ*^57^Fe = −0.24‰ for brain. For serum, the values were as follows: *Δ*^56^Fe = −0.30‰ and *Δ*^57^Fe = −0.30‰. No significant differences in the Cu or Zn isotopic composition in the brain or blood serum were observed between the L66 and NRMI-WT groups (Figs. 2 and 4).

For the 5xFAD and BL6-WT groups (Fig. 3 and Tables S3 and S4), no significant differences were established in terms of the Cu or Fe isotopic composition between mice, for neither the brain nor serum. Interestingly, the isotopic composition of Zn showed the trend of becoming lighter in the brain of 5xFAD mice (compared with BL6-WT), while opposite effects were seen for the respective isotopic compositions in the serum. The *δ*^67^Zn and *δ*^68^Zn values (*p* = 0.049 and *p* = 0.034, respectively *versus p* = 0.081 for *δ*^66^Zn) may indicate a lighter isotopic composition in the brain, while for serum, the *δ*^67^Zn values (*p* = 0.009) show an opposite tendency toward a heavier isotopic composition (depletion in the light ^64^Zn isotope). However, for *δ*^66^Zn and *δ*^68^Zn, *p* values were found to be 0.061 and 0.071, respectively. The absolute shift of isotopic composition for 5xFAD *versus* BL6-WT mice was as follows (brain/serum): *Δ*^66^Zn = −0.13/+0.09, *Δ*^67^Zn = −0.26/+0.17, *Δ*^68^Zn −0.43/+0.12‰.

**Figure 5:**
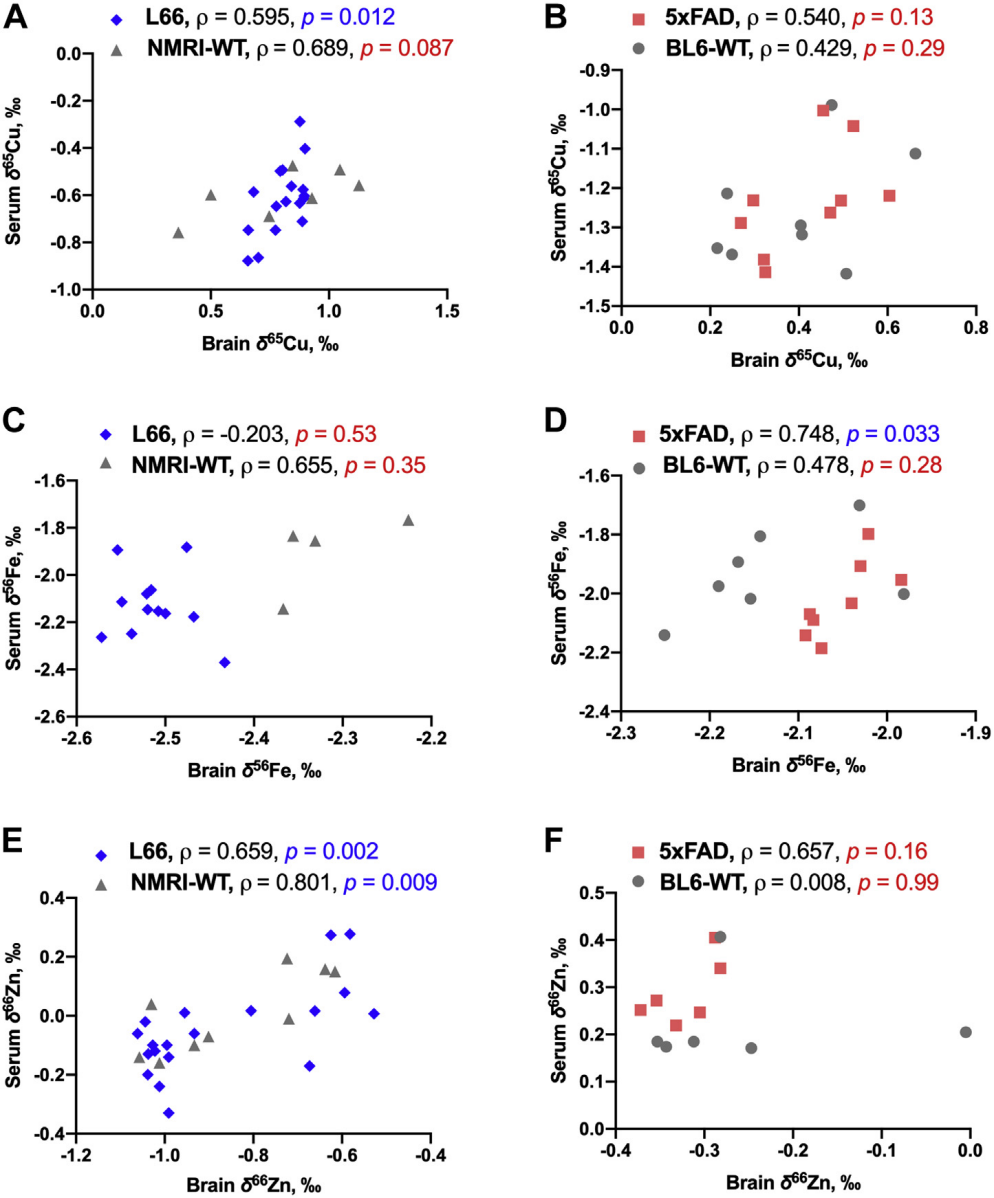
**Pearson’s correlations of *δ***^**65**^**Cu (*A* and *B*), *δ***^**56**^**Fe (*C* and *D*), and *δ***^**66**^**Zn (*E* and *F*) values between the brain and blood serum for L66 (*blue diamonds*) and NMRI-WT controls (*gray triangles*)—*A*, *C*, and *E*; for 5xFAD mice (*red square*) and matched BL6-WT (*gray circles*)—*B*, *D*, and *F***. Linear regressions were calculated by Pearson’s correlation and correlation coefficient *ρ* between pairs is given in the figure. Statistically significant correlations (*p* < 0.05) are indicated in blue font, while values that are not statistically significant are marked in red. All animals were males; the age of L66 and NMRI-WT mice was 11 to 12 months; the age of 5xFAD and BL6-WT animals was 5 to 6 months. The numbers of animals analyzed at the different research facilities are indicated in Table 1.

Next, we systematically compared Cu, Fe, and Zn isotopic compositions of the brain and serum (Fig. 5).

For NMRI-WT controls, we observed a positive correlation between the brain and serum data for the three isotope ratios *δ*^65^Cu, *δ*^56^Fe, and *δ*^66^Zn (Pearson’s R^2^ > 0.35). In L66, *δ*^65^Cu brain and serum values are likewise positively correlated (Pearson’s R^2^ > 0.45), but the overall *δ*^65^Cu value in L66 is lower than that in their WT controls (Fig. 5*A*). In L66, and contrary to NMRI controls, the *δ*^56^Fe values in the brain and serum, respectively, did not correlate (Fig. 5*C*, Pearson’s R^2^ = 0.04), while a similar positive correlation was seen for *δ*^66^Zn (Fig. 5*E*, Pearson’s R^2^ > 0.4). It should be noted though that only for *δ*^65^Cu in L66 mice and *δ*^66^Zn in both L66 and NMRI-WT animals, statistical significance of the correlation was reached at *p* < 0.05.

For both 5xFAD and BL6-WT mice (Fig. 5), we observed a positive and similar correlation, though weak, between the brain and serum *δ*^65^Cu values (Fig. 5*B*, Pearson’s R^2^ between 0.2 and 0.3). In BL6-WT mice, the *δ*^56^Fe values correlated fairly between the brain and serum (Fig. 5*D*, Pearson’s R^2^ > 0.2). However, in 5xFAD mice, this positive correlation for *δ*^56^Fe is stronger (Fig. 5*D*, Pearson’s R^2^ > 0.5, *p* < 0.05), and in general this element is isotopically lighter in 5xFAD than in BL6 controls (compare the slopes of 2.50 *versus* 0.75 for 5xFAD and BL6-WT mice, respectively, Fig. 5*B*). Brain and serum *δ*^66^Zn values (Fig. 5*F*) correlated positively in 5xFAD mice (Pearson’s R^2^ > 0.4), but not in BL6-WT mice (Pearson’s R^2^ < 0.0001). In this case, only the correlation of *δ*^56^Fe in 5xFAD mice reached statistical significance at *p* < 0.05.

## Discussion

High-precision isotopic analysis is an emerging approach for studying biochemical metal-related processes (34). For the lighter of any two isotopes, physicochemical processes proceed slightly faster (kinetic mass-dependent fractionation), while in chemical reactions, the heavier of any two isotopes has a slight preference for the strongest bonds (thermodynamic mass-dependent fractionation) at equilibrium (35). Biochemical processes may be accompanied by isotope fractionation, resulting in potential differences in the isotopic composition of a given metal between compartments. As biochemical processes are affected during disease processes, the isotopic composition of a metal in a given body compartment (*e.g.*, body fluid) may also be different in patients *versus* controls. Current analytical techniques, such as MC-ICP-MS, offer the precision required to reveal and quantify such isotope fractionation (36). High-precision isotopic analysis is being explored as a diagnostic tool for diseases that can otherwise only be established at a later stage and/or *via* more invasive techniques, or for obtaining a more profound insight into biochemical processes involving the element of interest (37, 38). So far, the isotopic composition of Cu was proven to be useful in the context of liver disease (39) and cancer (40, 41), that of Fe as a robust marker of individual Fe status, also in cases in which the currently used markers are no longer reliable (42, 43), and that of Zn in cancer (41, 44, 45). High-precision isotopic analysis has also been successfully applied in animal experiments to contribute to further insight into the factors governing the differences in isotopic composition (46, 47, 48).

Men and women with AD are known to exhibit different cognitive and psychiatric symptoms; women show a faster cognitive decline in AD and milder cognitive impairment (49). Such sex-dependent pattern is also reproduced in some *in vivo* models of AD (49, 50). This seems to correspond also to sex-based differences in the brain metal homeostasis (51). For instance, Maynard *et al.* (52) demonstrated significantly decreased Fe, Cu, and Zn levels in the brain of APP-overexpressing female mice, compared with males; but these sex-related differences were independent of APP/Aβ expression. Thus, to prevent such sex-related ambiguity, only male animals were investigated in the current study, which may be considered as a limitation since sex-based differences could not have been revealed.

In the current study, we observed *δ*^56^Fe and *δ*^57^Fe values that significantly differed when comparing results for the brains of tau (L66) and NMRI-WT mice. The results indicate that the brain tissue of mice under tau-pathology is enriched in the lighter Fe and Zn isotopes. Also in L66 serum, Fe was found to be isotopically lighter compared with the WT. These findings may indicate that Fe isotopic signatures in serum may show potential as a biomarker for tau-associated AD. Since the isotopic pattern of the elements in serum can be affected by the food intake, the animals’ chow was also analyzed (Table S1). As L66 mice exhibit acute neurological phenotype after the 10th month of life, they were supplemented with a protein-enriched chow for animal welfare reasons.

Notably, until the 10th month of life, both L66 and NMRI-mice were fed the same chow. *Post-hoc* analysis demonstrated no difference in the isotopic composition of Fe (*p* > 0.05) between the initial and the 10 to 12th month diet for L66 mice, which was implemented due to animal welfare reasons. However, for Cu and Zn, a significant difference in isotopic compositions (*p* < 0.01) was observed (Table S1). In principle, the difference (lighter isotopic composition) in the Cu and Zn serum isotopic composition between the L66-and NMRI-WT mice could therefore be (partly) related to the dietary change. Since the L66-mice received a different diet only at the end of the experiment, and the metal content was overall very similar, this is unlikely to dramatically affect the metal contents in the brain tissue. The intake between the two different diets varied by some milligrams per kilogram of the chow only (1 mg/kg for Cu, *ca*. +5%; 3 mg/kg for Zn, *ca*. −3%; and 9 mg/kg, *ca*. +5% for Fe). Importantly, for all elements under study, any significant effect of the diet change should not be anticipated. For Fe, there was no difference in isotopic composition between the two diets. For Cu, the observed (not statistically significant) trend toward a lighter isotopic composition in the brain and serum of L66 mice compared with NRMI-WT was the opposite of the dietary change as the 10 to 12th month chow was enriched in the heavier isotope (^65^Cu). Additionally, we evaluated the food consumption of the animals based on the body and brain weight at the time of sacrifice (Table S5). L66 mice had increased normalized brain weights (*p* < 0.001), accompanied by decreased body weight (*p* < 0.001), compared with the NRMI-WT. This is also confirmed for the 5xFAD model *versus* BL6-WT (*p* < 0.05). This observation may indicate reduced chow consumption in transgenic mice compared with the WTs, which might partially compensate for the effect of the consumption of the different chow by the L66 mice. Nevertheless, specifically, the data on Zn isotopic composition in L66 mice (which were also demonstrated to be not statistically significant) should be considered with care, and this can be considered an unavoidable limitation of the current study. The potential effect of the diet must be addressed in further research. For total quantification, significantly increased Fe contents and a tendency toward an increase in the level of Cu and Zn in L66 tau-transgenic mice compared with NMRI-WTs are consistent with previous reports for humans (for review see (14, 47)).

The 5xFAD mice and their matching BL6-WT controls were fed with the same chow for the whole duration of the experiment, and thus, the potential effect of the diet can be excluded for them. It is noteworthy that except for Cu, the isotopic compositions of Fe and Zn in the chow used to feed the 5xFAD and BL6-WT mice differed significantly (Table S1, *p* < 0.05) from those in both chows used to feed the L66 and NMRI-WT mice. Therefore, the differences observed between mouse lines may, at least partially, be attributed to a different nutritional baseline, as well as to a different genetic background. Unfortunately, proper comparison of the two WT lines is not possible due to different age, diet, and housing conditions. There was less Cu in brain tissue of the 5xFAD mice than in that of BL6-WT. Contrary to that, Zn was accumulated in the brain tissue of 5xFAD mice, which may be attributed to a dysregulated Zn homeostasis between the brain and blood (53). Since Aβ aggregation in the brain of these mice starts before 5 to 6 months of age, this could explain the lower Cu contents in the brain tissues of our 5-month-old 5xFAD transgenic mice.

For the 5xFAD mice, a lighter isotopic composition of Zn (*δ*^66^Zn, *δ*^67^Zn, and *δ*^68^Zn) was observed (Fig. 4). We observed statistical significance (*p* < 0.05) for *δ*^67^Zn and *δ*^68^Zn only. But as the *δ*^66^Zn value is not an independent variable from *δ*^67^Zn and *δ*^68^Zn due to the mass-dependent nature of the isotope fractionation, the finding for *δ*^66^Zn (not significantly different between the groups) may be related to the uncertainty of the measurements. The same may be suggested for the findings for Fe in serum for L66 *versus* NRMI-WT mice, where only for *δ*^56^Fe the significance level was reached. The difference in the trend for Fe isotopes between 5xFAD and L66 mice may indicate different biochemical pathways involved in changing iron homeostasis under different proteinopathies (54). In the case of tau-pathology in L66 mice, the lighter isotopic composition of Fe may be indicative of its increased turnover in the brain, which is probably not the case for the amyloidogenesis in 5xFAD mice (55). Additionally, the isotopic patterns in blood serum of 5xFAD mice *versus* BL6-WT were not found to display significant differences for Fe, while Zn was slightly enriched in the heavier isotopes in contrast to the corresponding brains. However, in this case the trend is even weaker due to very low levels of Zn in serum of 5xFAD and BL6-WT, leading to higher measurement uncertainty. Importantly, no significant difference in the Cu isotopic composition between transgenic mice and matched WT was found in both AD mouse models.

L66 tau transgenic mice show early-onset and extensive tau aggregation in multiple brain regions; these aggregates were reactive with silver and primulin, indicating the formation of stable tau aggregates (24). The tau pathology induces robust motor impairments in line with the symptomatology of FTD patients, such as abnormal gait pattern and dysfunction in motor coordination and motor learning (24). It is therefore not unexpected that metal homeostasis is severely impaired in these mice, also affecting the isotopic compositions of the elements considered. While this is important information for diagnostic considerations, it remains unclear whether it is the result of tau pathology or other disease processes and whether there may be a causal link.

The 5xFAD mouse model used in the current study is characterized by increased APP expression early in life, modeling familial AD, with pronounced, early amyloid pathology, neuronal loss (33, 56), and changes in spine density in the somatosensory and prefrontal cortex by ∼6 months of age (57). The age of onset is dependent on the genetic background. For the 5xFAD mouse on BL6/J background used in the current study, brain Aβ_42_ accumulation starts around 2 to 3 months of age (58). By 4 to 5 months, the animals exhibit neurological phenotype, including anxiety and freezing-fear behavior (58, 59). The 5xFAD mice develop congophilic amyloid angiopathy (60, 61), which also makes them an adequate model for human AD, often containing vascular pathology (62). Additionally, these mice, when kept on a C57BL6/J genetic background, exhibit epileptiform activity, independent of the presence of amyloid plaques, probably related to a high brain APP level *per se* (63). Recently, Bundy *et al.* (50) reported significant alteration of gene expression in 5xFAD female mice compared with matched WT by 4 months of age; many of the altered genes were found to be associated with immune function. Thus, by the age of 4 to 5 months, 5xFAD mice have considerable changes in brain physiology and biochemistry, which may affect metal homeostasis, *e.g.*, transition metal turnover and balance, and result in the differences in total levels of the elements and their isotopic composition as observed here.

Since the brain is strongly separated from the periphery by the blood–brain barrier (BBB), the chemical composition of blood serum as a peripheral fluid does not necessarily reflect the composition of the brain compartment (64, 65). To test whether serum isotopic signatures can be potential biomarkers for changes in brain metal homeostasis, we evaluated linear correlations of the *δ*-values between the brain and blood serum. Mostly not significant correlations were observed, except for *δ*^66^Zn in L66 *versus* NRMI mice, as well as for *δ*^65^Cu in L66 mice and for *δ*^56^Fe in x5FAD mice, but not for the matched WT controls in the case of Cu and Fe. Although the final number of points was rather low to be conclusive, the correlations revealed indicate that the serum isotopic composition is basically independent from that of the brain. This is in line with data for other potential biomarkers in AD (64, 66). The entrance of the metal ions and other nutrients to the brain, as well as the clearance of metabolites and toxins over the BBB, is strictly controlled in the healthy brain (67, 68). A wide spectrum of factors is probably involved in clearance and accumulation of the compounds in the brain *versus* blood (68), including transport mechanisms across the BBB, *i.e.*, passive diffusion *versus* active transport, sex, age, circadian rhythm, *etc*. Additionally, proteinopathies seem to create specific sinks for toxins such as metals, which may affect the equilibrium between tissues even further. Furthermore, metal ions seem to be able to hijack the corresponding transporters making them prone to accumulate in the brain tissue (69), while peripheral levels may remain low. Brain clearance of neurotoxic agents such as, first of all, Aβ and tau, but also other disease-implicated substances, is currently a major research area for both diagnostic and therapeutic advances in dementia research (70). The metals should be addressed more closely in this regard in further studies.

To the best of the authors’ knowledge, this is the first study reporting on the Fe isotopic compositions of blood serum and brain tissue in relevant AD models, as well as the first to report on the isotopic composition of Cu, Fe, and Zn in tau-transgenic mice. A previous study related to the isotopic composition of Zn as potentially relevant to AD was published in 2017 by Moynier *et al.* (71). They studied the isotopic signature of Zn in the brain tissue, red blood cells, and blood serum in 5xFAD compared with WT controls. The sampling was performed at 6, 9, and 12 months. Contrary to our study, the authors reported a heavier Zn isotopic composition in 5xFAD brains compared with that in the WT (significant at *p* < 0.01 for 12 months of age). Additionally, a change in the Zn isotopic composition with aging was also reported, which the authors attributed to the potential increase of free, nonprotein-bound Zn (71). In a follow-up study, the same group (72) reported isotope ratios for Cu in the blood serum and brain tissue of 5xFAD transgenic mice, sampled at the age of 3, 6, 9, and 12 months for the blood serum and at 9 and 12 months for the brain tissue, respectively. Importantly, the authors presented data for both sexes. Similar to our study, no significant difference in the Cu isotopic signature was detected between 5xFAD and WT mice (72). The individual *δ*-values (Supplementary Data) obtained here for the brain and blood serum were, generally, in line with the previous studies (46). Also, a significantly lighter Cu isotopic composition was observed in serum compared with that of the brain, which is in agreement with previously reported data (46, 72).

To conclude, the observed changes in the isotopic pattern of Fe and Zn in the brains and serum may be attributed to different pathological events in transgenic mice. Specific pathological processes in AD, such as deposition of misfolded protein aggregates of Aβ or hyperphosphorylated tau (54), are clearly accompanied by changes in the brain microenvironment, such as neuroinflammation. All these metals were shown to be modulating both Aβ and tau aggregation at several levels (10, 14, 73). Their biochemical activity regarding protein folding was rather widely explored in both *in vitro* and *in vivo* studies. For Aβ, those include affecting APP expression (74) and utilization by α-, β-, and γ-secretase (75), and direct binding of Aβ and its oligomers with free metal ions (54, 76). Cu, Fe, and Zn seem to be involved in tau-pathology by modulating the activity of cyclin-dependent kinase (CDK)5/p25 complex and glycogen synthase kinase-3β (GSK-3) (77, 78, 79) or affecting the activity of phosphatase like protein phosphatase 2A (PP2A) (80) and by binding tau *per se*. Metals, first of all, Fe and Zn, binding to Aβ and/or hyperphosphorylated tau might be, at least partially, responsible for the observations of the current study since such binding may induce mass-dependent isotope fractionation (38). However, this notion should be addressed in further research.

Another opportunity behind the current observation may be related to the modulation of the brain's metal intake. Common features of neurodegenerative disorders are the increased production of reactive oxygen species (81) and the decline of BBB and blood–cerebrospinal fluid barriers (67, 82), both of which may seriously impact the transition metal homeostasis (68). Critically, both animal (83) and human studies (84, 85) indicate the vulnerability of the neurovascular unit in AD (86), and both protective and trophic functions of the neural barrier seem to be impaired (82, 87). Simultaneously with altered metal–protein interactions due to the complexing capacities of amyloid-β and hyperphosphorylated tau, the deterioration of the barrier may be one of the reasons for the observed deviation of the isotopic patterns in AD murine models compared with matched WT mice. Reduced BBB integrity may promote excessive exposure of the brain to metal ions such as Fe and Zn leaking from serum proteins (54, 88), resulting in a shift in metal binding, releasing more “free” metal ions or, on the contrary, sequestering them from the normal biochemical turnover. That, in turn, may further exacerbate AD pathology. Metal homeostasis in early *versus* late dementia, the transport of metals *via* the BBB, and their accumulation profile associated with amyloid *versus* tau pathologies, should be addressed in future studies, as it may offer both diagnostic and therapeutic opportunities. Other prospects for further research would involve the study of sex- and age-based differences in isotopic signatures of neurodegeneration-associated metals in transgenic mice models and samples of AD patients, as well as the isotopic patterns in different brain compartments.

### Study strengths and limitations

We consider the use of relevant transgenic mice models of AD and high-precision isotopic analysis as well as interlaboratory comparisons applied as the strengths of our study. However, this study has certain limitations: different numbers of animals per group were used; for L66 mice a different chow was administered at the end of the of the study due to animal welfare requirements; Fe level and isotopic composition were assessed in one laboratory only, which resulted in a lower number of observations for these parameters; the study was conducted at one fixed age of the animals and with male individuals only, thus, providing no information on potential sex- and age-based effects; finally, Aβ or tau-protein was not assessed in this study.

## Experimental procedures

### Mice and tissue

Experiments on animals were carried out in accordance with the European Communities Council Directive (63/2010/EU) with local ethical approval, *i.e.*, either a project license issued under the UK Scientific Procedures Act 1986 (PPL 60/4085, for 5xFAD and BL6-WT mice), or in accordance with the German Law for Animal Protection (Tierschutzgesetz; G0068/18, for Line 66 and NMRI-WT mice).

#### Tau-transgenic mice

Male homozygous tau-transgenic L66 and NMRI wild-type controls were generated as previously described by Melis *et al.* (24). Two aggregation-promoting mutations, P301S and G335D in the repeat domain, were inserted into the tau cDNA and L66 mice were bred and maintained on an NMRI background. Mice were bred in pressurized isolators and pathogen-free conditions. They were then colony-housed (up to four per cage) in Type 2 Macrolon wire lid cages on corn cob bedding in a controlled facility (temperature 20–22 °C, 60–65% humidity, air changes: 17–20 changes per hour). The animals were under a 12-h light/dark cycle and had *ad libitum* access to food and water. In this study, 26 L66 and 14 NMRI-WT mice at 11 to 12 months of age at the time of sacrifice were used (Table 1). Both mouse lines were housed at Charité and received the same chow (V1534-3; metal levels according to the manufacturer, confirmed locally by ICP-MS: 16, 176, and 94 mg/kg for Cu, Fe, and Zn, respectively) for the first 10 months. After 10 to 12 months, when L66 mice developed a considerable tremor, the standard chow had to be substituted with a different chow with higher protein content (V1124-3; metal levels: 17, 185 and 91 mg/kg for Cu, Fe, and Zn, respectively) for ethical reasons.

#### APP/PSEN1 transgenic mice

Male homozygous 5xFAD-transgenic (5xFAD) and their C57BL6/J wild-type (BL6-WT) littermates were generated as previously described (32). 5xFAD mice were bred and maintained on a C57BL6/J background. Mice were kept in a holding room with a 12-h light/dark cycle, the temperature was maintained at 23 °C ± 2 °C and relative humidity was 40 to 60%. Mice were allowed *ad libitum* access to food and water (metal levels according to the manufacturer, confirmed locally by ICP-MS: 16, 131, and 87 mg/kg for Cu, Fe, and Zn, respectively). Twenty 5xFAD and 20 BL6-WT mice (∼5 months of age at the time of sacrifice) were used in this study (Table 1). These mice were housed at the University of Aberdeen and received the same chow during the whole experimentation period.

#### Perfusion of mice and collection of brain tissue and blood serum

Mice were injected intraperitoneally with Euthatal as anesthetic at a dose volume of 10 ml/kg of body weight (5XFAD and BL6-WT) or with ketamine/xylazine (0.2 ml of 100 mg/ml ketamine, 0.2 ml of 20 mg/ml xylazine, and 0.6 ml of 0.9% saline) at a dose volume of 6 ml/kg of body weight (L66 and NMRI-WT). Blood was collected *via* cardiac puncture in lithium-heparin-tubes and the mice were perfused *via* intracardiac puncture with heparinized saline solution (50 mg of heparin per litre of 0.9% saline) for 3 min before harvesting the brains. The brain was separated into hemispheres, transferred into Eppendorf vials, and immediately frozen in liquid nitrogen. Blood was centrifuged for 5 min at 2000*g* in reaction tubes after standing for 20 to 30 min. The serum was then transferred into Eppendorf tubes and snap-frozen in liquid nitrogen. Brain and serum samples were kept at −80 °C until use. All containers used for sampling were acid and ultrapure water washed in cleanroom conditions to avoid metal contamination. The body and the brain weights of all mice were taken at the time of sacrifice. Body weight (g), brain weight (mg), and normalized brain weight (brain weight to body weight ratio (mg/g)) are given in Table S5.

### Analytical methods

The study was conducted at multiple centers. An overview of the study details, including mouse housing facilities, and a description of which laboratories involved carried out quantitative determination and isotopic analysis of Fe, Cu, and Zn, is provided for both sample groups in Table 1.

### Sample preparation

The analysis of the brain tissue and blood serum was performed at three separate analytical facilities (at BAM, Ghent University, and University of Aberdeen) to ensure data quality. For BAM, all sample preparation steps (except for the digestion step) were carried out in an ISO 6 clean room (PicoTrace); the digestion system and the ICP-MS instruments are located in ISO 7 clean rooms. For Ghent University, all sample manipulations were performed in an ISO 4 clean room (PicoTrace). At the University of Aberdeen, all sample preparation steps were carried out in analytical chemistry labs under a laminar flow hood. For quantitative determination of the metal contents, the sample preparation protocol consisted of the mineralization of the sample (serum and brain tissue) *via* acid digestion. For isotopic analysis, additional sequential chromatographic separation of the target analytes from the matrix is required. The measurements were performed using SF-ICP-MS or Q-ICP-MS and MC-ICP-MS for element contents and isotope ratios, respectively. Animal chow (0.1 g per replicate) was prepared and analyzed analogously with the brain tissue to evaluate the background isotopic composition of the animals.

#### Digestion procedure

BAM: In 10-ml quartz vessels, the digestion was accomplished using 3.2 ml and 2.5 ml conc. HNO_3_ (67–70%, purified in-house by two-stage subboiling distillation) for the brain (whole) and serum samples, respectively. A high-pressure asher system (HPA-S, Anton Paar, Austria) was used for sample digestion. The operating conditions for the HPA-S were: ramping to 300 °C over 30 min and holding at 300 °C for another 90 min and then allowing to cool down. Pressure was set to 100 bar throughout the digestion program. The contents of the digestion vessel were transferred to a 15-ml PFA vessel (Savillex). The digestion vessel was thoroughly rinsed with 0.28 mol/l HNO_3_ and the rinse was transferred to the PFA vessel. The digestion solution was evaporated till dryness at 120 °C. The dried residue was dissolved in 1 to 2 ml conc. HCl (32–35%, purified in house by two-stage subboiling distillation) and then dried. This process was repeated and the residue was finally redissolved in 8 mol/l HCl +0.001% H_2_O_2_ for the chromatographic separation of Cu, Fe, and Zn from the sample matrix.

Ghent University: Ultrapure water (resistivity ≥18.2 MΩ cm at 25 °C) was obtained from a Milli-Q Element water purification system (Merck Millipore). Trace metal analysis grade 14 mol/l HNO_3_ and 12 mol/l HCl (PrimarPlus, Fisher Chemicals) were further purified by subboiling distillation in a Savillex DST-4000 acid purification system (Savillex Corporation). The purified acids thus obtained were titrated prior use to establish the exact concentration. TraceSELECT 9.8 mol/l H_2_O_2_ acquired from Sigma-Aldrich was used for sample preparation. Brain tissue, animal chow, and blood serum samples were digested using a mixture of subboiled 14 mol/l HNO_3_ and 9.8 mol/l H_2_O_2_ in Teflon Savillex beakers at 110 °C for 16 h. Four milliliter of HNO_3_ and 1 ml of H_2_O_2_ was used for the brain tissue or chow specimens; 2 ml of HNO_3_ and 0.5 ml of H_2_O_2_ were used for blood serum digestion. Subsequently, the sample digests were evaporated to dryness at 90 °C and redissolved in 5 ml of 8 mol/l HCl containing a small amount of H_2_O_2_ (∼0.001%) to assure occurrence of Cu and Fe in the Cu(II) and Fe (III) oxidation states, respectively, for the chromatographic isolation of the target elements. The samples were used for isotopic analysis only.

University of Aberdeen: Mouse brains were separated into hemispheres prior to digestion. Each hemisphere was 200 to 400 mg in weight and was digested in 1.5 ml of ultrapure concentrated (65%) nitric acid using a microwave system (Ethos Up, Analytix). Samples were digested in TMF inserts, using a predetermined program with a maximum temperature of 200 °C being maintained for 20 min and subsequently cooled to <35 °C. Following digestion, samples were diluted to 10 g with ultrapure Milli-Q water. The samples were used for total quantification only.

#### Target element isolation

For the determination of isotope ratios at both BAM and Ghent University, Fe, Cu, and Zn were chromatographically separated from the matrix *via* a protocol modified from that developed and reported by Lauwens *et al.* (89) and Van Heghe *et al.* (90) using strong anion exchange resin. Analytical grade AG MP-1 strong anion exchange resin (100–200 μm dry mesh size, chloride anionic form, Bio-Rad) packed in polypropylene chromatographic columns (Bio-Rad PolyPrep) was used.

The separation procedure is shown in Table S6. Spectral and nonspectral interferences from concomitant matrix elements were thus virtually eliminated. The purified Fe, Cu, and Zn fractions were evaporated till dryness and the residues redissolved twice in 14 mol/l HNO_3_ to remove residual chlorides. The final residue was redissolved in 0.28 mol/l HNO_3_ for determination of the total element content and isotope ratios. Quantitative recoveries were obtained for the three elements upon chromatographic separation (∼100%), thus ensuring that potential on-column isotope fractionation would not affect the final isotope ratio data.

### Determinations by ICP-MS

#### Total element content

BAM: The total contents of Cu, Zn, and Fe in the brain and serum digests were determined *via* external calibration using a single-collector sector field ICP-MS unit (Element 2/Element XR, Thermo Scientific), operated at medium mass resolution (R ≈ 4000). The instrument was equipped with a jet interface and a sample introduction system consisting of a 200 μl min^−1^ quartz concentric nebulizer and a cyclonic spray chamber. Tuning, mass calibration, and determination of mass offsets of the target isotopes were performed before each analytical sequence. In brief, the samples and calibration standards were prepared in 0.28 mol/l HNO_3_ and Ge (50 μg/l) was used as an internal standard to correct for matrix effects and instrument instability. The instrument settings and data acquisition parameters are presented in Table S7. The analytical method was validated using NIST SRM 1598a (Inorganic Constituents in Animal Serum, NIST) and Seronorm Trace Element Serum level 1 (Sero AS).

University of Aberdeen: The total contents of Fe, Cu, and Zn in the brain digests were determined using an Agilent 7900 quadrupole-based ICP-MS instrument using no gas mode for Cu and Zn and hydrogen gas mode for Fe. During analysis, a 10 μg/l Y and 10 μg/l Rh internal standard solution was continuously introduced into the system. The instrument settings and data acquisition parameters are presented in Table S7.

#### Isotope ratios

Single-element standard stock solutions of Cu, Fe, Zn, Ni, and Ga (Inorganic Ventures) were used for element quantification and mass bias correction. The following isotopic reference materials were used for external mass bias correction: NIST SRM 976 (National Institute of Standards and Technology) for Cu, IRMM-014 (European Commission) for Fe, and IRMM-3702 (European Commission) for Zn. Single-element standard solutions of Cu, Fe, and Zn (Inorganic Ventures), previously characterized for their isotopic composition (in-house standard solutions), were used for monitoring the quality of the isotope ratio measurements. All standards and samples were properly diluted with 0.28 mol/l HNO_3_ for elemental determination and isotope ratio measurement.

Isotope ratio measurements were accomplished using a Neptune multicollector (MC)-ICP-MS instrument (Thermo Scientific) at both BAM and Ghent University. The instrument settings were tuned daily (Table S8). The measurements were performed at (pseudo) medium mass resolution, in static collection mode and using 10^11^ Ω amplifiers connected to the Faraday collectors. Samples were measured in a sample-standard bracketing approach (SSB).

For SSB, Ghent University used the following isotopic reference materials: NIST SRM 976 for Cu, IRMM-014 for Fe, and IRMM-3702 for Zn. The internal standards used for correction of instrumental mass discrimination were Ni, Ga, and Cu for the Fe, Cu, and Zn isotope ratio measurements, respectively. Correction for instrumental mass discrimination was performed using a combination of internal correction by means of the revised Russell’s law (91) and external correction using the isotopic reference materials cited above.

BAM used BAM RS standards as bracketing standard for Cu and Zn. The BAM RS standards were then characterized relative to the isotopic reference materials NIST SRM 976 (Cu, purchased as IRMM-633 from LGC Standards GmbH, Germany) and IRMM-3702 (Zn). The delta values obtained relative to the BAM RS standards were then recalculated relative to NIST SRM 976 and IRMM-3702, as has been described by Vogl *et al.* (92).

The isotope ratios were expressed in delta notation (*δ*, per mil, ‰) relative to the respective isotopic reference material and determined using Equations 1, 2 and 3 for Cu, Fe, and Zn, respectively.

(1) δ65Cu=((C65u/C63u)sample(C65u/C63u)standard−1)

(2) δyFe=((Fye/F54e)sample(Fye/F54e)standard−1)

(3) δyZn=((Zyn/Z64n)sample(Zyn/Z64n)standard−1)

in which *y* is 56 or 57 (for the ^56/54^Fe or ^57/54^Fe isotope ratios) or 66, 67, or 68 (for the ^66/64^Zn, ^67/64^Zn, or ^68/64^ Zn isotope ratios).

### Interlaboratory validation

A rigorous interlaboratory comparison scheme was executed for quality assurance/quality control (QA/QC). For the total element content, Seronorm Trace Element Serum level 1 (traceable to NIST SRMs, Sero, Norway) was used as QC sample by BAM and the University of Aberdeen where the validation of the analytical method was performed following the FDA guidelines (93). All recoveries were in agreement with the ±20% acceptability criteria of the FDA guidelines for method validation (Tables S9 and S10).

BAM and Ghent University were supplied with a QC sample of blood serum, provided by LGC Ltd for isotopic analysis. The QC sample was analyzed independently and blindly in accordance to the sample analysis protocol used for the real samples. Acceptable agreement was obtained for the Cu, Fe, and Zn isotope ratios (Tables S11 and S12) between BAM and Ghent University.

### Statistical analysis

Data analysis was performed using SPSS Statistics 23 (IBM Corp) and Prism 8 (GraphPad Software). For each group of mice, median *δ*-values were calculated and used to evaluate statistical differences between each transgenic model and its respective control group (5xFAD *versus* BL6-WT and L66 *versus* NMRI-WT). The outliers were identified using a Grubbs' test (*α* = 0.05) and excluded from further data evaluation. The normality of the distribution was tested using a Shapiro–Wilk test (*α* = 0.05) for each group of mice. The difference between the groups based on data obtained in several laboratories (namely, total levels of Cu and Zn in brain, isotopic composition of Cu and Zn, except Zn in blood serum of 5xFAD and BL6-WT mice) was assessed using analysis of covariance (ANCOVA) to exclude the effect of the measurement facility. For the data obtained in a single laboratory only (total Fe in the brain, isotopic composition of Fe, and isotopic composition of Zn in the blood serum of 5xFAD and BL6-WT mice), an unpaired *t*-test or Mann–Whitney rank test was used for normally distributed (parametric) and for nonnormally distributed (nonparametric) data sets, respectively. A level of *p* < 0.05 was considered as statistically significant.

The correlation between the brain and serum isotopic composition was evaluated using Pearson’s equation; the correlation coefficient (*ρ*) and the *p*-value were calculated. Only the subjects for whom the data for both brain tissue and blood serum were available, were included in the correlation analysis.

In case of a statistically significant difference or potential trend in isotopic composition, the *Δ*-values were calculated as follows:

(4) ΔC65u=(median[δC65utestinggroup]−median[δC65umatchedcontrol])

(5) ΔFye=(median[δFyetestinggroup]−median[δFyematchedcontrol])

(6) ΔZyn=(median[δZyntestinggroup]−median[δZynmatchedcontrol])

in which *y* is 56 or 57 (for the Fe isotope ratios) or 66, 67, or 68 (for the Zn isotope ratios). The use of *Δ*-values is a conventional approach for the data analysis in isotopic geochemistry (94), which was already applied to biological samples as well (95, 96).

## Data availability

Raw data for elemental quantification and isotopic analysis, including statistical processing, are presented in a supplementary data file. All remaining data are contained within the article and supplementary tables.

## Conflict of interest

The authors declare no conflict of interest.
