# Supplementary material for: Cu, Fe, and Zn isotope ratios in murine Alzheimer's disease models suggest specific signatures of amyloidogenesis and tauopathy
Source: J Biol Chem. 2021 Jan 14;296:100292. doi: 10.1016/j.jbc.2021.100292 (PMC7949056; doi:10.1016/j.jbc.2021.100292)
Supplement: Supplemental Tables S1–S11 [file mmc1.pdf]

Supplementary information

Cu, Fe and Zn isotope ratios in murine Alzheimer's disease models suggest specific signatures ofamyloidogenesis and tauopathy

Nikolay Solovyev[1§¶], Ahmed H. El-Khatib[2,3§], Marta Costas-Rodríguez[1], Karima Schwab[4], Elizabeth Griffin[5], Andrea Raab[5,6], Bettina Platt[4], Franz Theuring[7], Jochen Vogl[2], Frank Vanhaecke[1*]

[1]Ghent University, Department of Chemistry, Atomic & Mass Spectrometry–A&MS research unit, Campus Sterre, Krijgslaan 281-S12, 9000 Ghent, Belgium
[2]BAM Bundesanstalt für Materialforschung und –prüfung, Richard-Willstätter-Str. 11, 12489 Berlin, Germany
[3]Department of Pharmaceutical Analytical Chemistry, Faculty of Pharmacy, Ain Shams University, African Union Authority St., Abbassia, Cairo, Egypt
[4]Institute of Medical Sciences, School of Medicine, Medical Sciences & Nutrition, Foresterhill, University of Aberdeen, Aberdeen, Scotland, AB25 2ZD, United Kingdom
[5]Trace Element Speciation Laboratory (TESLA), Department of Chemistry, University of Aberdeen, Aberdeen, Scotland, AB24 3UE, United Kingdom
[6]Institute of Chemistry, Environmental Analytical Chemistry, University of Graz, Universitätsplatz 1, 8010 Graz, Austria
[7]Charité – Universitätsmedizin Berlin, Institute of Pharmacology, Hessische Str. 3-4, 10115 Berlin, Germany
[§] Both authors contributed equally to this work.
[¶] Current address: Institute of Technology Sligo, Ash Lane, F91 YW50 Sligo, Ireland

*Corresponding author: Frank Vanhaecke: Ghent University, Department of Chemistry, Atomic & Mass Spectrometry–A&MS research unit, Campus Sterre, Krijgslaan 281-S12, 9000 Ghent, Belgium,
Tel: +32 9 264 48 48
E-mail: frank.vanhaecke@ugent.be

Table S1. Isotopic compositions of the animal chow, expressed relative to NIST SRM 976, IRMM 014 and IRMM 3702 for Cu, Fe and Zn, respectively (SD – standard deviation).

δ-value ± SD, ‰	L66 (V1124-3)[a]	NMRI-WT (V1534-3)	5xFAD / C57BL6 /J[b]
$\delta\,^{65}Cu$	0.13 ± 0.05**	0.05 ± 0.04	0.15 ± 0.05
$\delta\,^{56}Fe$	-0.08 ± 0.14	-0.05 ± 0.13	0.17 ± 0.10
$\delta\,^{57}Fe$	-0.09 ± 0.21	-0.06 ± 0.20	0.23 ± 0.17
$\delta\,^{66}Zn$	-0.59 ± 0.07**	-0.41 ± 0.07	-0.04 ± 0.07
$\delta\,^{67}Zn$	-0.89 ± 0.15**	-0.58 ± 0.15	-0.05 ± 0.17
$\delta\,^{68}Zn$	-1.16 ± 0.10**	-0.81 ± 0.11	-0.09 ± 0.11

[a] L66 mice received the same chow (V1534-3) as NMRI-WT for the first 10 months of the experiment. Between 10 and 12 months L66 mice were switched to V1124-3 with a higher protein content to improve their health status.

[b] Both mouse lines obtained the same chow during the whole duration of the experiment.

Table S2.

Cu, Fe and Zn contents (*w*) in brain tissue of L66 (A) and 5xFAD mice (B) *vs*. matched WT-controls. Total element contents were measured by SF-ICP-MS/Q-ICP-MS. The distribution of the data was tested for normality using a Shapiro-Wilk test at $p<0.05$. All animals were males; the age of the animals was 11-12 months and 5-6 months for A and B, respectively. The numbers of animals analysed at the different research facilities are indicated in **Table 1** of the manuscript.

A: L66 *vs*. NMRI-WT							
Parameter	Line	Mean	Median	SD	Range (min/max)[a]	*p*-value for the difference between the lines	*n* [b]
*w*Cu, mg/kg	L66	5.79	5.81	0.25	5.44 / 6.20	0.071 [c]	23
	NMRI	5.64	5.66	0.21	5.24 / 5.95		10
*w*Fe, mg/kg	L66	15.68	16.14	2.16	12.45 / 18.20	0.014 [d]	12
	NMRI	12.84	12.58	0.92	11.89 / 13.89		5
*w*Zn, mg/kg	L66	12.34	12.36	1.53	10.00 / 16.18	0.455 [c]	25
	NMRI	11.93	12.01	1.25	9.63 / 14.31		11

B: 5xFAD *vs*. BL6-WT							
Parameter	Line	Mean	Median	SD	Range [a]	*p*-value between lines	*n* [b]
*w*Cu, mg/kg	5xFAD	3.70	3.70	0.20	3.32 / 4.07	0.029 [c]	18
	BL6	3.84	3.88	0.15	3.55 / 4.10		14
*w*Fe, mg/kg	5xFAD	13.04	12.65	1.11	11.98 / 14.57	0.987 [d]	8
	BL6	13.02	13.15	2.41	9.79 / 17.46		9
*w*Zn, mg/kg	5xFAD	12.82	13.14	0.98	11.10 / 13.89	0.001 [c]	13
	BL6	11.79	12.47	1.34	9.75 / 13.40		17

a – minimum to maximum value range.
b – number of animals excluding the outliers (please, see supplementary data file for more details); the outliers were removed based on a Grubbs' test ($p < 0.05$).
c – ANCOVA for the data from two independent laboratories.
d – unpaired *t*-test for the data from a single laboratory.

Table S3.

Cu, Fe and Zn isotopic compositions of brain tissue in L66 (A) and 5xFAD BL6-WT mice (B) *vs*. matched WT-controls. The isotopic composition of the elements in the brain was measured by MC-ICP-MS. The distribution of the data was tested for normality using a Shapiro -Wilk test at $p<0.05$, indicating that the distribution was not normal. All animals were males; the age of the animals was 11-12 months and 5-6 months for A and B, respectively. The numbers of animals analysed at the different research facilities are indicated in **Table 1** of the manuscript.

A: L66 *vs*. NMRI-WT

Parameter	Line	Mean	Median	SD	Range (min/max)^{a}	*p*-value for the difference between the lines	n^{b}
δ^{65}Cu, ‰	L66	+0.80	+0.80	0.09	+0.66 / +0.95	0.509 ^{c}	24
	NMRI	+0.75	+0.85	0.29	+0.31 / +1.13		11
δ^{56}Fe, ‰	L66	-2.51	-2.52	0.04	-2.57 / -2.43	0.0001 ^{d}	13
	NMRI	-2.33	-2.36	0.06	-2.39 / -2.23		5
δ^{57}Fe, ‰	L66	-3.70	-3.70	0.05	-3.79 / -3.63	0.0001^{d}	13
	NMRI	-3.44	-3.46	0.10	-3.53 / -3.27		5
δ^{66}Zn, ‰	L66	-0.83	-0.87	0.19	-1.06 / -0.53	0.752 ^{c}	26
	NMRI	-0.82	-0.72	0.17	-1.06 / -0.62		11
δ^{67}Zn, ‰	L66	-1.21	-1.27	0.30	-1.57 / -0.77	0.521 ^{c}	26
	NMRI	-1.21	-1.05	0.26	-1.58 / -0.91		11
δ^{68}Zn, ‰	L66	-1.62	-1.65	0.40	-2.09 / -0.98	0.622 ^{c}	26
	NMRI	-1.61	-1.41	0.35	-2.07 / -1.16		11

B: 5xFAD *vs*. BL6-WT

Parameter	Line	Mean	Median	SD	Range ^{a}	*p*-value between lines	n^{b}
δ^{65}Cu, ‰	5xFAD	+0.55	+0.56	0.18	+0.27 / +0.94	0.627 ^{c}	20
	BL6	+0.55	+0.54	0.20	+0.22 / +0.87		18
δ^{56}Fe, ‰	5xFAD	-2.06	-2.07	0.05	-2.13 / -1.98	0.273 ^{d}	9
	BL6	-2.10	-2.13	0.10	-2.25 / -1.96		10
δ^{57}Fe, ‰	5xFAD	-3.03	-3.04	0.08	-3.15 / -2.88	0.327 ^{d}	9
	BL6	-3.08	-3.12	0.13	-3.27 / -2.90		10
δ^{66}Zn, ‰	5xFAD	-0.18	-0.21	0.16	-0.39 / +0.10	0.081 ^{c}	20
	BL6	-0.14	-0.08	0.16	-0.39 / +0.09		20
δ^{67}Zn, ‰	5xFAD	-0.32	-0.38	0.22	-0.56 / +0.04	0.049 ^{c}	19
	BL6	-0.21	-0.13	0.24	-0.70 / +0.07		20
δ^{68}Zn, ‰	5xFAD	-0.42	-0.58	0.29	-0.77 / +0.02	0.034 ^{c}	19
	BL6	-0.30	-0.15	0.31	-0.78 / +0.03		20

a – minimum to maximum value range.
b – number of animals excluding the outliers (please, see supplementary data file for more details); the outliers were removed based on a Grubbs' test ($p < 0.05$).
c – ANCOVA for the data from two independent laboratories.
d – Mann-Whitney rank test for the data from a single laboratory.

Table S4.

Cu, Fe and Zn isotopic compositions of blood serum in L66 (A) and 5xFAD BL6-WT mice (B) *vs*. matched WT-controls. The isotopic composition of the elements in the serum was measured by MC-ICP-MS. The distribution of the data was tested for normality using a Shapiro -Wilk test at $p<0.05$, indicating that the distribution was not normal. All animals were males; the age of the animals was 11-12 months and 5-6 months for A and B, respectively. The numbers of animals analyzed at the different research facilities are indicated in **Table 1** of the manuscript.

A: L66 *vs*. NMRI-WT							
Parameter	Line	Mean	Median	SD	Range (min/max)[a]	*p*-value between lines	*n* [b]
δ^{65}Cu, ‰	L66	-0.60	-0.61	0.16	-0.88 / -0.28	0.951 [c]	22
	NMRI	-0.60	-0.60	0.09	-0.76 / -0.48		8
δ^{56}Fe, ‰	L66	-2.13	-2.15	0.14	-2.37 / -1.88	0.019 [d]	13
	NMRI	-1.90	-1.84	0.17	-2.14 / -1.77		4
δ^{57}Fe, ‰	L66	-3.07	-3.03	0.22	-3.48 / -2.73	0.082 [d]	13
	NMRI	-2.79	-2.73	0.20	-3.06 / -2.63		4
δ^{66}Zn, ‰	L66	-0.04	-0.07	0.17	-0.34 / +0.33	0.186 [c]	25
	NMRI	+0.04	+0.04	0.15	-0.16 / +0.28		9
δ^{67}Zn, ‰	L66	+0.03	-0.08	0.41	-0.74 / +1.12	0.356 [c]	25
	NMRI	-0.11	-0.14	0.40	-0.85 / +0.37		7
δ^{68}Zn, ‰	L66	-0.03	-0.14	0.38	-0.55 / +0.81	0.652 [c]	
	NMRI	-0.09	-0.15	0.28	-0.49 / +0.44		
B: 5xFAD *vs*. BL6-WT							
Parameter	Line	Mean	Median	SD	Range [a]	*p*-value between lines	*n* [b]
δ^{65}Cu, ‰	5xFAD	-1.27	-1.25	0.18	-1.64 / -1.00	0.850 [c]	10
	BL6	-1.26	-1.30	0.14	-1.42 / -0.99		9
δ^{56}Fe, ‰	5xFAD	-2.03	-2.06	0.12	-2.19 / -1.80	0.182 [d]	8
	BL6	-1.93	-1.97	0.15	-2.14 / -1.70		6
δ^{57}Fe, ‰	5xFAD	-2.94	-3.00	0.18	-3.15 / -2.64	0.201 [d]	8
	BL6	-2.82	-2.92	0.23	-3.13 / -2.45		6
δ^{66}Zn, ‰	5xFAD	+0.30	+0.27	0.07	+0.22 / +0.41	0.061 [d]	8
	BL6	+0.23	+0.18	0.09	+0.17 / +0.41		5
δ^{67}Zn, ‰	5xFAD	+0.53	+0.46	0.19	+0.35 / +0.87	0.009 [d]	9
	BL6	+0.34	+0.29	0.13	+0.22 / +0.62		5
δ^{68}Zn, ‰	5xFAD	+0.59	+0.48	0.27	+0.34 / +1.13	0.071 [d]	9
	BL6	+0.42	+0.36	0.17	+0.29 / +0.77		5

a – minimum to maximum value range.
b – number of animals excluding the outliers (please, see supplementary data file for more details); the outliers were removed based on a Grubbs' test ($p < 0.05$).
c – ANCOVA for the data from two independent laboratories.
d – Mann-Whitney rank test for the data from a single laboratory.

Table S5. Data on body weight, brain weight and normalised brain weight (brain weight/body weight ratio) for the animals used in the current study. The data are presented as mean (median) ± standard deviation.

Mice line	Number of animals	Body weight, g	Brain weight, mg	Normalised brain weight, mg/g	*p*-value*
L66	26	28.7 (28.3) ± 2.1	475 (475) ± 20	16.6 (16.9) ± 0.9	<0.001
NMRI-WT	11	48.7 (48.5) ± 3.9	498 (502) ± 11	10.3 (10.3) ± 0.7	
5xFAD	20	25.6 (25.4) ± 3.6	488 (481) ± 25	19.3 (19.7) ± 2.2	<0.05
BL6-WT	20	29.4 (30.4) ± 3.7	470 (468) ± 20	16.3 (15.6) ± 2.3	

* Mann-Whitney rank test

Table S6. Sequential chromatographic protocol for Cu, Fe and Zn isolation from the sample matrix using AG® MP-1 resin.

**A: Ghent University**		
**Separation step**	**1 mL resin – Serum**	**2 mL resin – Brain tissue, animal chow**
	**Medium/eluent**	**Medium/eluent**
Cleaning	5 mL Milli-Q water	5 mL Milli-Q water
	3 mL 7 mol/L HNO_3	7 mL 7 mol/L HNO_3
	10 mL Milli-Q water	10 mL Milli-Q water
	10 mL 0.7 mol/L HNO_3	10 mL 0.7 mol/L NO_3
	10 mL Milli-Q water	10 mL Milli-Q water
Conditioning	5 mL 8 mol/L HCl + 0.001% H_2O_2	10 mL 8 mol/L HCl + 0.001% H_2O_2
Sample load	5 mL 8 mol/L HCl + 0.001% H_2O_2	5 mL 8 mol/L HCl + 0.001% H_2O_2
Matrix elution	3 mL 8 mol/L HCl + 0.001% H_2O_2	8 mL 8 mol/L HCl + 0.001% H_2O_2
Cu elution	9 mL 5 mol/L HCl + 0.001% H_2O_2	12 mL 5 mol/L HCl + 0.001% H_2O_2
Fe elution	7 mL 0.7 mol/L HCl	10 mL 0.7 mol/L HCl
Zn elution	7 mL 0.7 mol/L HNO_3	10 mL 0.7 mol/L HNO_3

**B: BAM**	
**Separation step**	**2 mL resin – Serum & Brain**
	**Medium/eluent**
Cleaning	5 mL Milli-Q water
	3 mL 7 mol/L HNO_3
	10 mL Milli-Q water
	10 mL 0.7 mol/L HNO_3
	10 mL Milli-Q water
	10 mL Milli-Q water
	12 mL 5 mol/L HCl
	10 mL Milli-Q water
Conditioning	10 mL 8 mol/L HCl + 0.001% H_2O_2
Sample load	5 mL 8 mol/L HCl + 0.001% H_2O_2
Matrix elution	8 mL 8 mol/L HCl + 0.001% H_2O_2
Cu elution	12 mL 5 mol/L HCl + 0.001% H_2O_2
Fe elution	10 mL 0.7 mol/L HCl
Zn elution	12 mL 0.7 mol/L HNO_3

Table S7. Instrument settings and data acquisition parameters for total element content quantification in brain samples and in animal chow by ICP-MS.

**A: BAM**	
**Instrument settings**	Element 2 single collector sector field ICP-MS
RF power (W)	1250
Sampler cone	Ni, 1 mm id
Skimmer cone	Ni, 0.5 mm id
Lens settings	Optimized for maximum analyte signal intensity and stability
Ar flow-rates (L min^{-1})	Plasma 16; auxiliary 0.90; nebulizer 1.160
Sample uptake rate (µL min^{-1})	200
Resolution mode	Medium (R ≈ 4000)
Acquisition mode	ASX-100 autosampler
**Data acquisition parameters**	
Mass window	125%
Sample time	0.01 s
Samples per peak	10
Segment duration	0.13 s
Search window	100%
Integration window	80%
Nuclides monitored	Cu method: ^{63}Cu, ^{65}Cu, ^{72}Ge
	Zn method: ^{64}Zn, ^{66}Zn, ^{72}Ge
**B: University of Aberdeen**	
Instrument	Agilent 7900 ICP-MS
Sample introduction	Agilent SPS 4 Autosampler + PeriPump
Nebulizer	MicroMist
Uptake Speed (Nebulizer pump)	0.1 rps
Spray chamber	Scott double-pass
Nebulizer gas flow rate	1.16 L min^{-1}
Plasma gas flow rate	12 L min^{-1}
ISTD	Y, Rh 0.1 rps uptake
ICP RF power	1550 W
Cones	Ni
Reaction Gas Modes	Cu and Zn: No gas. Fe: H_2
Nuclides monitored	^{56}Fe, ^{57}Fe, ^{63}Cu, ^{65}Cu, ^{64}Zn, ^{65}Cu, ^{66}Zn, ^{67}Zn

Table S8. Instrument settings and data acquisition parameters for isotope ratio measurements in brain and serum samples by MC-ICP-MS.

**A: Ghent University**	
**Instrument settings**	
RF power (W)	1200
Guard electrode	Connected
Sampler cone	Ni, Jet-type, 1.1 mm aperture diameter
Skimmer cone	Ni, H-type, 0.8 mm aperture diameter
Lens settings	Optimized for maximum analyte signal intensity
Ar flow rates (L min^{-1})	Plasma 15; auxiliary 0.80; nebulizer 1.0-1.1
Sample uptake rate (µL min^{-1})	100
Resolution mode	(pseudo) medium (RP ~ 4000)
**Data acquisition parameters**	
Acquisition mode	Static, multi-collection
Number of blocks	9
Number of cycles	5
Integration time (s)	4.194
Cup configurations	L3: ^{63}Cu; L2: ^{64}Zn; L1: ^{65}Cu; C: ^{66}Zn; H1: ^{67}Zn; H2: ^{68}Zn<br>L4: ^{54}Fe; L2: ^{56}Fe; L1: ^{57}Fe; C: ^{58}(Fe+Ni); H1: ^{60}Ni; H3: [illegible]<br>L4: ^{63}Cu; L2: ^{65}Cu; C: ^{67}Zn; H2: ^{69}Ga; H4: ^{71}Ga
**B: BAM**	
**Instrument settings**	
RF power (W)	1250
Guard electrode	Connected
Sampler cone	Ni, standard
Skimmer cone	Ni, H-type
Lens settings	Optimized for maximum analyte signal intensity
Ar flow-rates (L min^{-1})	Plasma 16; auxiliary 0.7-0.8; nebulizer 1.05-1.10
Sample uptake rate (µL min^{-1})	100
Resolution mode	(pseudo) medium (RP ~ 4000)
**Data acquisition parameters**	
Acquisition mode	Static, multi-collection
Number of blocks	1
Number of cycles	50
Integration time (s)	4.194
Cup configurations	L3: ^{63}Cu; C: ^{65}Cu<br>L4: ^{62}Ni; L2: ^{64}Zn; C: ^{66}Zn; H2: ^{67}Zn; H3: ^{68}Zn

Table S9. Total contents of Fe, Cu and Zn of the Seronorm™ Trace Elements Serum L-1 (human); data shown are measured *vs.* certified concentrations with expanded uncertainty U (k=2) and recovery %. Data obtained by BAM using Element 2 single collector sector field ICP-MS.

	**Fe**		**Cu**		**Zn**	
	measured	certified[1]	measured	certified[2]	measured	certified[3]
Value (mg/L)	1.34	1.47	1.05	1.09	1.16	1.10
U	0.15	0.30	0.11	0.09	0.13	0.15
Recovery %	91		97		105	

[1]Traceable to NIST SRM 3126a, [2]Traceable to NIST SRM 3114, [3]Traceable to NIST SRM 3168a

Table S10. Total contents of Fe, Cu and Zn of the Seronorm™ Trace Elements Serum L-1 (human); data shown are measured *vs.* certified concentrations with expanded uncertainty U (k=2) and recovery %. Data obtained by University of Aberdeen using Agilent 7900 quadrupole ICPMS.

	**Fe**		**Cu**		**Zn**	
	measured	certified[1]	measured	certified[2]	measured	certified[3]
Value (mg/L)	1.74	1.47	1.02	1.09	1.10	1.10
U	0.12	0.30	0.08	0.09	0.14	0.15
Recovery %	118		94		100	

[1]Traceable to NIST SRM 3126a, [2]Traceable to NIST SRM 3114, [3]Traceable to NIST SRM 3168a

Table S11. Isotopic composition of Fe, Cu and Zn of the prospective human serum reference material "Serum-LGC"; data shown are delta values (‰) with expanded uncertainty *U* (*k*=2), expressed relative to IRMM-014, NIST SRM 976, and IRMM-3702 for Fe, Cu, and Zn, respectively. Data obtained by Ghent University.

Uncertainty u_c was calculated from uncertainty budget as follows:

$$u_c = \sqrt{u_{internal}^2 + u_{external}^2 + u_{sample\ preparation}^2 + u_{isotopic\ standard}^2}$$

	δ^{56} Fe	δ^{57} Fe	δ^{65} Cu	δ^{66} Zn	δ^{67} Zn	δ^{68} Zn
Value, ‰	-2.11	-3.09	-0.20	+0.00	-0.03	-0.02
u_c	0.15	0.22	0.08	0.10	0.22	0.15
U	0.30	0.44	0.15	0.19	0.45	0.29

Table S12. Isotopic composition of Fe, Cu and Zn in the prospective human serum reference material "Serum-LGC"; data shown are delta-values (‰) with expanded uncertainty U (k=2), expressed relative to IRMM-014, IRMM-633, and IRMM-3702 for Fe, Cu and Zn, respectively. Data obtained by BAM.

Uncertainty u_c was calculated from uncertainty budget as follows:

$$u_c = \sqrt{u_{sample}^2 + u_{standard1}^2 + u_{standard2}^2 + u_{sample\ preparation}^2 + u_{reproducibility}^2}$$

	δ^{56} Fe	δ^{57} Fe	δ^{65} Cu	δ^{66} Zn	δ^{67} Zn	δ^{68} Zn
Value, ‰	-2.00	-2.78	n/a *	+0.36	+0.56	+0.73
u_c	0.11	0.23	n/a *	0.16	0.24	0.32
U	0.22	0.46	n/a *	0.32	0.48	0.64

* Sequence failed
